# Prognostic Value of GIMAP4 and Its Role in Promoting Immune Cell Infiltration into Tumor Microenvironment of Lung Adenocarcinoma

**DOI:** 10.1155/2022/7440189

**Published:** 2022-10-06

**Authors:** Siyuan Chen, Dong Tian, Lauren Petersen, Shuchang Cao, Zachary Quinn, Junyan Kan, Mingfeng Zheng, Wenjun Mao, Yuan Wan

**Affiliations:** ^1^Department of Cardiothoracic Surgery, The Affiliated Wuxi People's Hospital of Nanjing Medical University, Wuxi, Jiangsu 214023, China; ^2^The Pq Laboratory of BiomeDx/Rx, Department of Biomedical Engineering, Binghamton University, Binghamton, New York 13902, USA; ^3^Department of Thoracic Surgery, West China Hospital, Sichuan University, 37 Guo Xue Xiang, Chengdu 610041, China; ^4^Department of Biomedical Engineering, Pratt School of Engineering, Duke University, Durham, North Carolina 27708, USA

## Abstract

GIMAPs are recognized as an important regulator in the carcinogenesis and development of lung cancer, but the function of GIMAP4 in the tumor microenvironment (TME) of lung cancers is unclear. In this study, we investigated the expression and variation of GIMAP4 in lung adenocarcinoma (LUAD), to explore its association with infiltration of immune cells. The Cancer Genome Atlas (TCGA) data and Gene Expression Omnibus (GEO) data were analyzed. Infiltration of immune cells was identified with TIMER (Tumor Immune Estimation Resource) and TISIDB (an integrated repository portal for tumor-immune system interactions). GIMAP4 expression declined in non-small-cell lung cancer (NSCLC), correlated with a poor overall survival (OS) in LUAD, indicating that GIMAP4 was a promising prognostic biomarker in LUAD. GIMAP4 mutation frequency was 1.76% in TCGA cohort and was relevant to the expression of immune components. TIMER and CIBERSORT analysis further confirmed that high GIMAP4 expression possibly promoted immune cell infiltration into the TME, with low GIMAP4 impairing the efficacy of immunotherapies targeting common immune check point inhibitors (ICI). GO (Gene Ontology) and KEGG (Kyoto Encyclopedia of Genes and Genomes) analyses were performed to provide insights into biological processes involved in LUAD. GIMAP4 was expected to be a prognostic biomarker in LUAD and provides potential adjuvant or neoadjuvant therapeutic strategies for targeting ICIs.

## 1. Introduction

Despite significant improvements in survival rates due to the early diagnosis using low-dose computed tomography and innovative application of tyrosine kinases inhibitors [1], lung cancer remains the leading cause of cancer death worldwide [2]. Early diagnosis and effective intervention are crucial [3, 4], and thus, biomarkers for selection of patients and treatment monitoring are needed in clinical settings. Particularly, efficient biomarkers for immunotherapy are highly desirable but unavailable yet. Recent studies reveal the role of tumor microenvironment (TME) in carcinogenesis and disease progression [5]. Moreover, accumulating evidence suggests that tumor-infiltrating immune cells (TIC), including B cells, dendritic cells, and T cells, are intimately involved in the development of lung cancer [6–8]. Simultaneously, therapeutic strategy involving immune stimulant led to remarkable prolonged survival against diverse lung cancer cases. Therefore, the understanding of dynamic TME and profiles of tumor infiltrating immune cells may lead to discovery of efficacy biomarkers and new strategies for immunotherapy, e.g., immune checkpoint therapy [9, 10].

GTPase of immunity-associated proteins (GIMAP) are extensively expressed in the immune system, which engage in early Th cell differentiation. GIMAP was also found to be correlated with the immune components of the TME. In GIMAP family, GIMAP4 is rarely investigated, and much about its function(s) remains unknown. Recent studies imply that GIMAP4 is involved in Th cell secretory processes [11]. In cervical cancer, the prognostic potential of GIMAP4 has been identified, and the immunoscore of TICs is strongly related to GIMAP4 [12]. Moreover, in breast cancer, GIMAP4 might be a protective factor [13]. However, there was limited knowledge regarding roles of GIMAP4 in NSCLC, including its expression, pathological features, survival, and prognosis [14]. In this study, we identified gene mutations and differentially expressed genes (DEG) that verify differences in GIMAP4 expression between lung cancers and normal lung tissues. We found its related pathway contributes to the tumor immune response. We primarily ascertained its function on immune cell infiltration and immune response in lung adenocarcinoma (LUAD). Immune checkpoints (ICP) CD274 (PDL1), PDCD1 (PD1), CTLA4, and LAG3 were noted positively correlated with GIMAP4 expression, and further pathways under immune checkpoint inhibitors (ICIs) were explored. GIMAP4 participated in T cells activation based on our findings through GSEA. Eventually, an immune landscape in LUAD was constructed based on gene expression and distribution in the local microenvironment. Our results direct to a complex tumor immune microenvironment and provide the theoretical basis for immunotherapy of next generation.

## 2. Materials and Methods

### 2.1. Work Flow of Current Work

**Figure 1:**
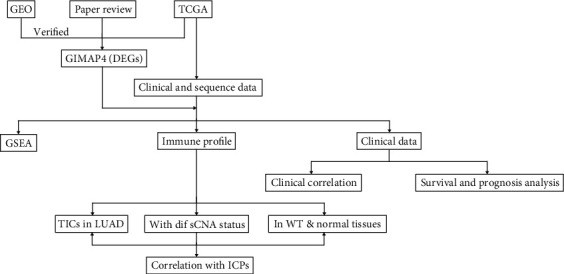
The analysis workflow of this study. Abbreviation: DEGs: differentially expressed genes; GSEA: gene set enrichment analysis; TICs: tumor-infiltrating immune cells; sCNA: somatic copy-number alteration; WT: wild type; ICPs: immune checkpoints; LUAD: lung adenocarcinoma.

RNA-seq profiles from TCGA and GEO were used to identify differential expression of GIMAP4 in LUAD, which was targeted as grouping and sorting basis for subsequent analysis. Parallel results were gained in TCGA survival data, indicating a relationship between GIMAP4 expression and tumor staging. ESTIMATE algorithm was employed to calculate immunoscore as an alternative separating factor. DMGs were identified based on the median GIMAP4 expression. GSEA-based GO and KEGG analyses were conducted. Simultaneously, CIBERSORT and TIMER algorithms were employed to analyze TICs. Finally, correlations between GIMAP4 expression and several cytokines and between GIMAP4 expression and immune checkpoints were obtained using Spearman correlation test, respectively. The whole workflow of our work was presented in Figure 1.

### 2.2. TCGA and GEO Cohort Analysis of GIMAP4 Expression

Totally, 513 lung adenocarcinoma (LUAD) positive and 59 LUAD negative samples were collected from The Cancer Genome Atlas (TCGA) database [15]. The same screening process was carried out in patients diagnosed with lung squamous cell carcinoma (LUSC), with 501 diseased samples and 49 normal ones. Statistical analyses were performed using R software package ggplot2. The statistical significance was tested by log rank test, and the significant threshold of *p* value was set to 0.05. GIMAP4 expression in protein aspect was displayed using THPA dataset (https://www.proteinatlas.org/). 116 samples were screened from the GEO database in GSE32863 dataset in format MINIML with 58 LUAD samples and 58 normal tissue [16]. Meanwhile, we downloaded 35 LUSC RNA-seq samples and 28 paracancerous tissue samples in GSE12472 dataset to conduct differential analysis [17]. Box plot of GIMAP4 expression was drawn by R software package ggpuber.

### 2.3. Survival Analysis

RNA-seq data for 513 samples and corresponding clinical information were downloaded from TCGA. The criteria for exclusion are as follows: (1) normal samples; (2) samples with a survival time shorter than one month; (3) samples with incomplete information. Information was analyzed, and a scatter plot of gene expression was produced using R package (ggrisk). Survival and survminer packages were employed for creation of KM curves. The timeROC package was utilized to construct time-dependent ROC of GIMAP4. All analytical methods and R packages were performed using R software version v4.0.3. A *p* value less than 0.05 was considered as statistically significant.

### 2.4. Clinical Bioinformatics Verifying GIMAP4 Mutations in LUAD

Lollipop plots, oncoplots, and cohort summary plots were used to display mutation distribution, somatic landscape, and, distribution of variants, which can indicate a high mutation frequency of GIMAP4 in LUAD (https://www.aclbi.com/static/index.html#/). Totally, 513 patients were enrolled with pathological diagnosis confirmed to be NSCLC with stage I-IIIA. We analyzed mutation, transcription, and clinical data to identify the somatic mutation rate of LUAD patients. Mutation data was downloaded and visualized using “maftools” package in R software [18].

### 2.5. Identification of DEGs

DEGs were identified by differential analysis via LinkedOmics (http://www.linkedomics.org/login.php) [19]. Heatmaps and volcano plot were made to visualize gene regulation. Gene Ontology (GO) and Kyoto Encyclopedia of Genes and Genomes (KEGG) analyses were performed with Gene Set Enrichment Analysis (GSEA), aiming to reveal characteristic pathways in LUAD by searching relevant upstream and downstream genes.

### 2.6. Tumor Immune Estimation Resource (TIMER) Analysis

Infiltration of B cells, CD4+, CD8+, neutrophil, and macrophage cells in LUAD patients was performed using TIMER (http://timer.cistrome.org/#) [20]. GIMAP4 expression in tumors and healthy tissue was compared in Exp model. Changes of immune infiltration between tumors with GIMAP4 mutated and wild type (WT) were performed in violin plots, as was correlation between immune infiltration level and different somatic copy-number alteration (sCNA) status of GIMAP4.

### 2.7. Analysis of TICs

Samples acquired were identified by comparing high- and low- immunity cohorts using package limma [21]. CIBERSORT was then loaded to evaluate the proportion of TICs profile in LUAD samples [22]. A *p* value less than 0.05 was considered statistically significant.

### 2.8. TISIDB Database

TISIDB database (http://cis.hku.hk/TISIDB/index.php) was used to explore the correlation between GIMAP4 expression and immune subtypes in LUAD or LUSC [23]. Spearman correlation between GIMAP4 expression and receptors, immunoinhibitors, immunostimulators, chemokines, MHC molecules, and lymphocytes was visualized using heatmaps.

### 2.9. Analysis of ICPs

The data were downloaded from TCGA. The multigene correlation map was constructed with the pheatmap package. We used Spearman's correlation analysis to visualize relationship between quantitative variables without a normal distribution. A *p* value less than 0.05 was considered statistically significant.

## 3. Results

### 3.1. GIMAP4 Expression Declined in LUAD

**Figure 2:**
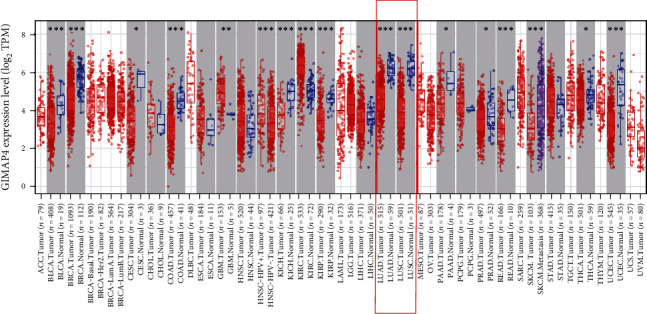
GIMAP4 expression in tumors versus adjacent normal tissue. ^∗^*p* < 0.05, ^∗∗^*p* < 0.01, ^∗∗∗^*p* < 0.001. GIMAP4 is significantly expressed higher in NSCLC than adjacent normal tissue.

GIMAP4 expression in tumors versus adjacent normal tissues is shown in Figure 2.

Significant changes of GIMAP4 were showed in breast cancer, cervical squamous cell carcinoma, LUAD, LUSC, etc.

**Figure 3:**
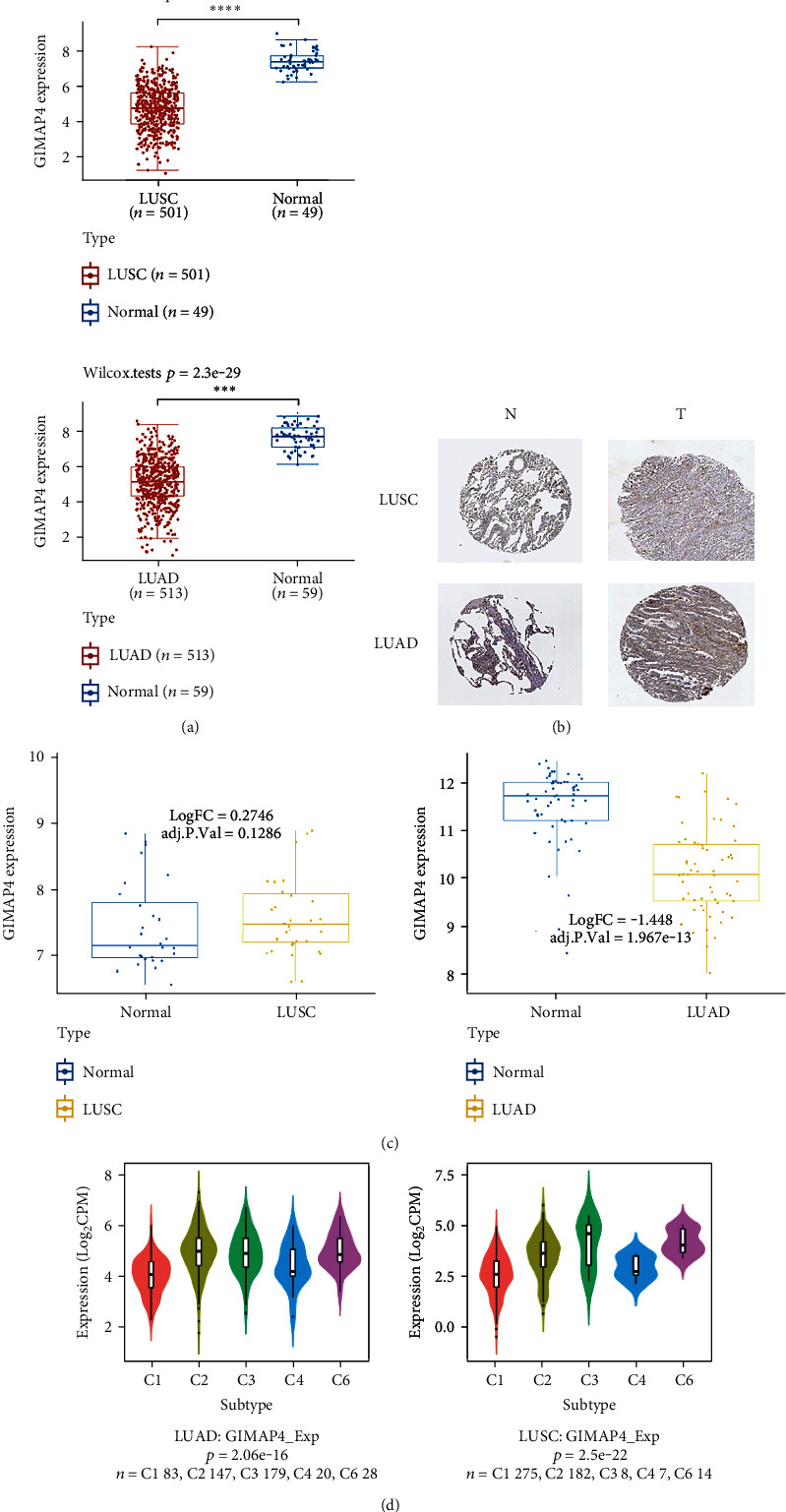
GIMAP4 expression in NSCLC subtypes. (a) Expression of GIMAP4 in lung adenocarcinoma (LUAD) and in lung squamous cell carcinoma (LUSC). (b) Expression of GIMAP4 in lung tissue. (c) Verification of GIMAP4 expression in GEO database. (d) Association between GIMAP4 and immune subtypes of lung cancers. C1: wound healing; C2: IFN-gamma dominant; C3: inflammatory; C4: lymphocyte depleted; C5: immunologically quiet; C6: TGF-b dominant.

The expression level of GIMAP4 was significantly downregulated in clinical LUAD specimens compared to adjacent noncancerous lung tissue (Figure 3(a)). Significant decline of GIMAP4 protein expression (Figure 3(b)) in both LUAD and LUSC tissue were observed compared with normal lung tissue. From GEO datasets, LUAD set GSE32863, and LUSC set GSE12472 were used to verify GIMAP4 expression. It is noteworthy that the fold change in expression between LUSC and adjacent normal lung samples did not reach statistical significance (Figure 3(c)). Given the heterogeneity of LUSC and the intrinsic limitation of online public data, additional investigation into the role of GIMAP4 in LUSC is required. GIMAP4 expression across different immune subtypes (C1: wound healing, C2: IFN-*γ* dominant, C3: inflammatory, C4: lymphocyte depleted, C6: TGF-*β* dominant) of LUAD and LUSC was elucidated in Figure 3(d). Considering the aim of current work, subtype C5 (immunologically quiet) was excluded.

Significant differences in GIMAP4 expression were observed among five immune subtypes (wound healing, IFN-gamma dominant, inflammatory, lymphocyte depleted, TGF-b dominant), implying potentially diverse function of GIMAP4 during separate immunologic processes.

### 3.2. Diminishing GIMAP4 Levels Correlates with Poor OS in LUAD but Not in LUSC

**Figure 4:**
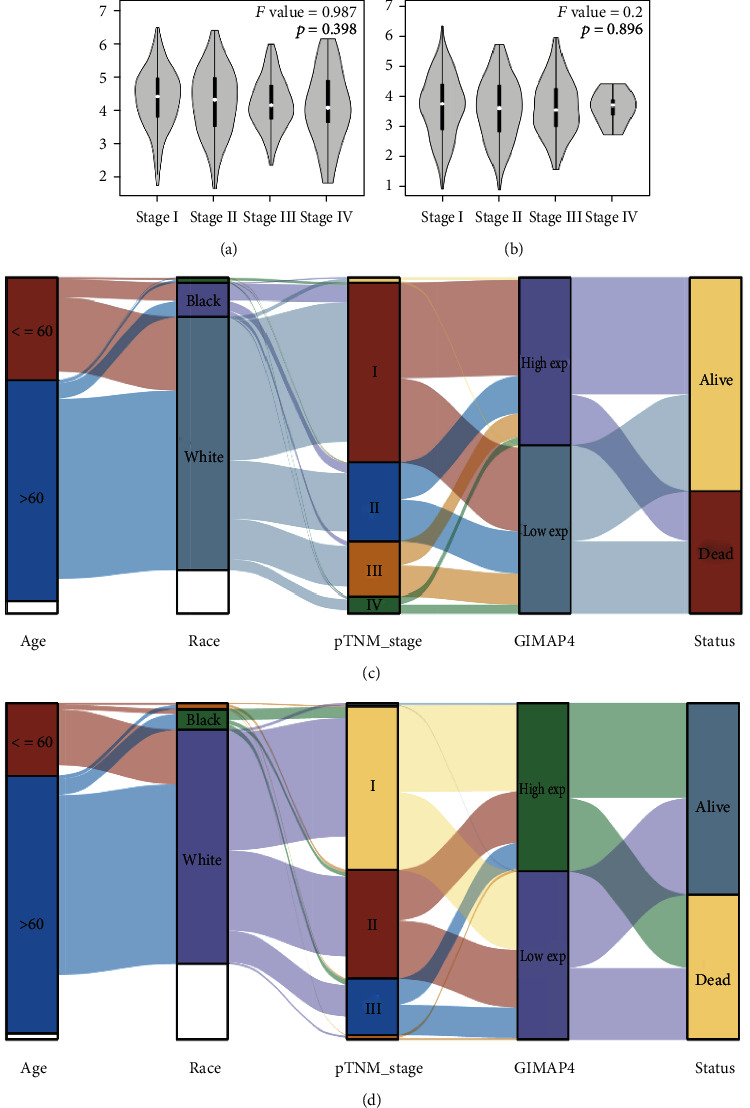
GIMAP4 expression among different clinical stages and clinical characteristics. Correlation between GIMAP4 expression and cancer stages in (a) LUAD and (b) LUSC. Correlation between GIMAP4 expression and clinical characteristics in (c) LUAD and (d) LUSC.

GIMAP4 expression did not differ significantly among disparate stages in LUAD (Figure 4(a)) or LUSC (Figure 4(b)). Sankey diagrams detailing correlation between GIMAP4 expression and clinical characteristics in LUAD and LUSC were constructed (Figures 4(c) and 4(d)). GIMAP4 in stage I LUAD patients seemed with little difference. However, with grade increased, patients tended to have lower expression of GIMAP4 and manifested worse prognosis. In contrast, parallel relation between TNM stage, GIMAP4 expression, and prognosis was not observed in LUSC.

**Figure 5:**
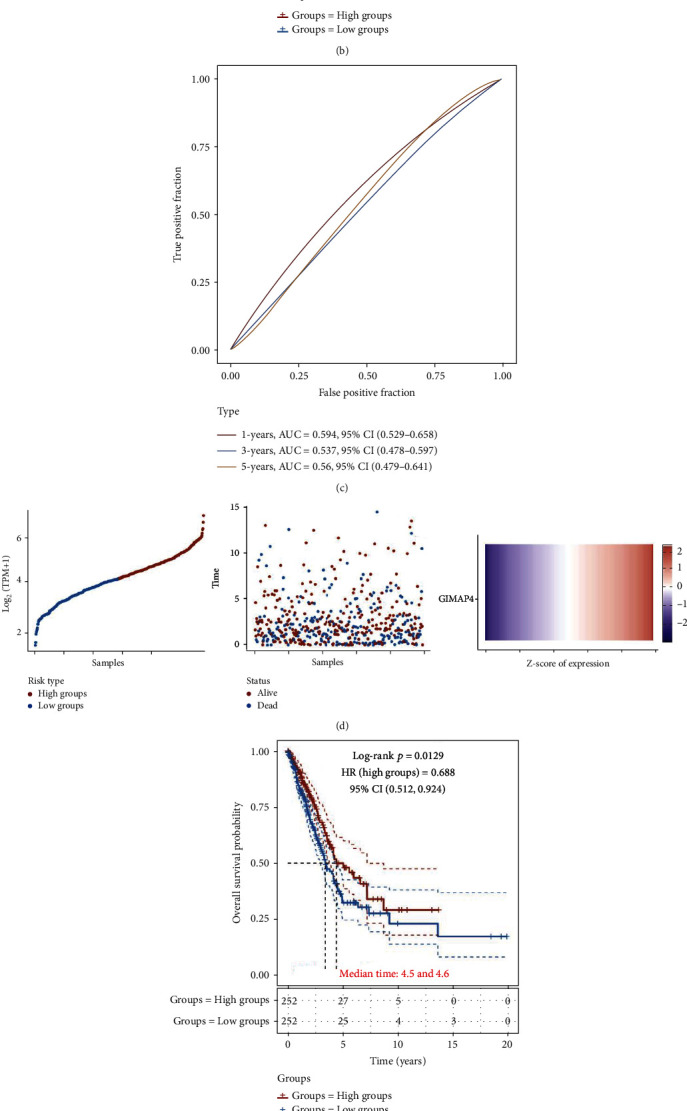
Prognostic analysis of GIMAP4 in LUAD and LUSC samples using TCGA set. (a, d) The left is a scatter plot of gene expression from low to high. The middle is the scatter distribution of survival time and survival status of different sample genes. The right is a heatmap of GIMAP4 expression. (b, e) Kaplan-Meier survival analysis of GIMAP4 in 1, 3, and 5 years. (c, f) Time-dependent ROC analysis of the GIMAP4.

Analysis of gene expression, survival time, and survival status of different sample genes suggested a correlation between GIMAP4 upregulation and longer patient survival (Figure 5(a)). As such, LUAD patients with lower GIMAP4 exhibited worse OS (Figure 5(b)). Time-dependent ROC analysis of GIMAP4 level was exhibited in Figure 5(c). The predictive ability of GIMAP4 was positively correlated with AUC value, indicating GIMAP4 as a promising prognostic biomarker in LUAD. Similar trends were present in LUSC patients (Figures 5(d) and 5(f)), though no significant difference in OS was observed (Figure 5(e)), which implied limited prognostic potential of GIMAP4 in LUSC. Consequently, we focused on the potential biological and immunologic role of GIMAP4 in LUAD.

### 3.3. Identification of GIMAP4 Mutations in LUAD

**Figure 6:**
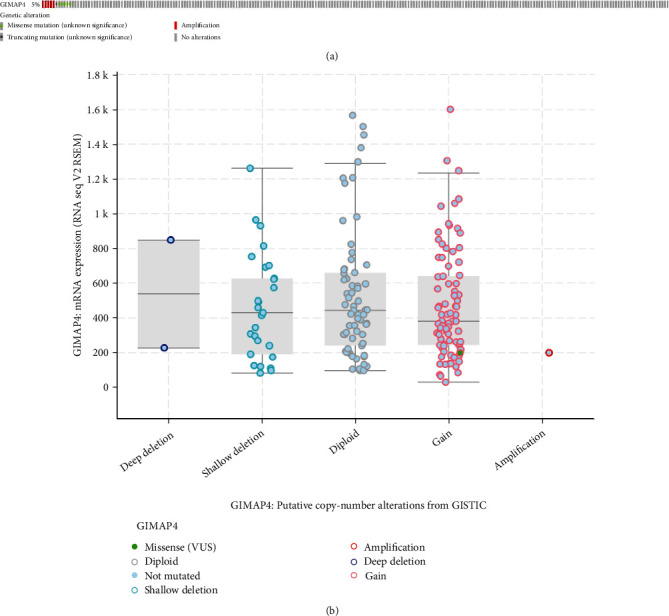
Genetic alterations profiling of GIMAP4 in NSCLC according to The Cancer Genome Atlas. (a) Genetic alterations in LUAD and LUSC patients. Box plots of GIMAP4 expression in (b) lung adenocarcinoma (LUAD) and (c) lung squamous cell carcinoma (LUSC) based on the genetic status for LUAD and LUSC.

*GIMAP4* mutations occurred in 5% LUAD cases, containing missense mutation and amplification in majority (Figure 6(a)). *GIMAP4* gain and shallow deletion were predominant in LUAD as evidenced by *GIMAP4* copy numbers (Figure 6(b)).

**Figure 7:**
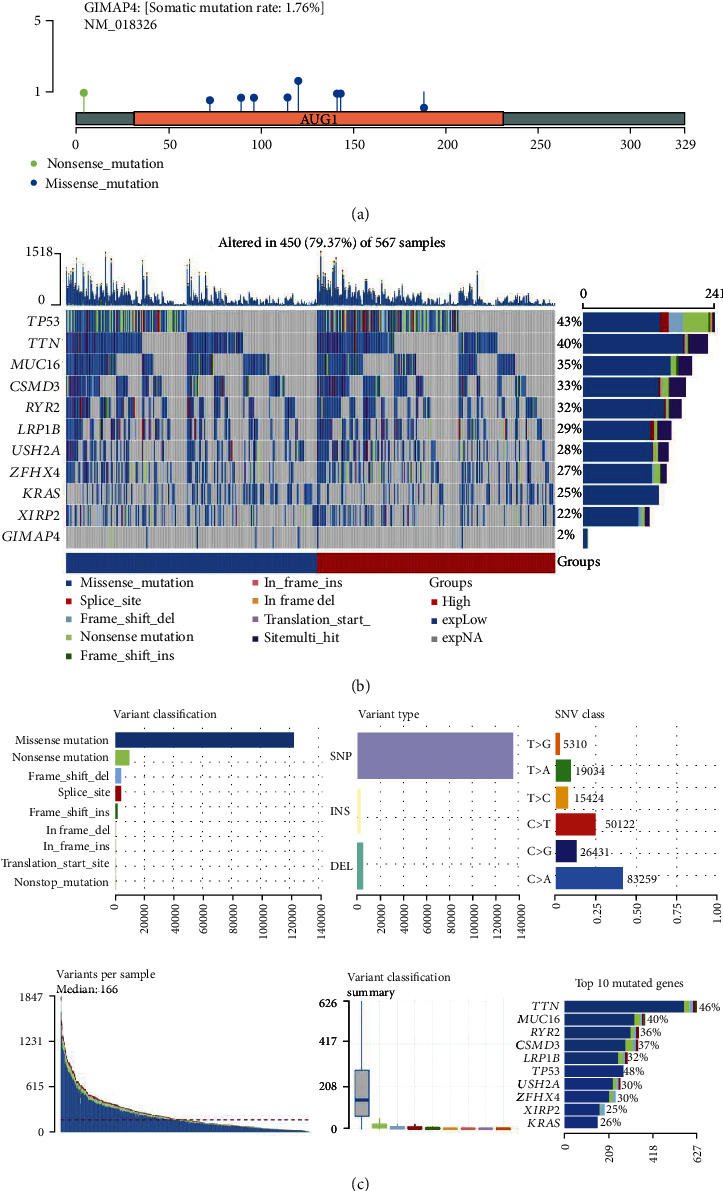
Mutation landscape of LUAD across different expression of GIMAP4. (a) Lollipop plot displaying mutation distribution and protein domains for GIMAP4 in cancer with labeled recurrent hotspots. Somatic mutation rate and transcript names are indicated by plot title and subtitle. (b) Oncoplot displaying the somatic landscape of LUAD cohort. Genes are ordered by their mutation frequency, and samples are ordered by GIMAP4 expression as indicated by the annotation bar. Side bar plot shows log10 transferred *Q*-values estimated by MutSigCV. Mutation information of each gene in each sample was shown in the waterfall plot, where different colors with specific annotations (bottom) meant the various mutation types. The bar plot above exhibited the number of mutation burden. (c) Cohort summary plot displays distribution of variants based on variant classification, type, and SNV class. Plots at the bottom (from left to the right) indicate mutation load per sample, variant classification type, and ten mutated genes.

Lollipop plots showed that the somatic mutation rate of *GIMAP4* was 1.76% and mainly in coding region (Figure 7(a)). To identify any correlations between gene mutation and immune component, we keenly investigated differences in the genetic layer between high- and low-immunity cohorts. The resulting waterfall plot displayed the somatic landscape of the LUAD cohort with genes ordered by their mutational frequency and samples ordered by GIMAP4 expression (Figure 7(b)). Mutations of known oncogenes, including TP53, accumulated in samples with low level of GIMAP4. What is more, other mutated genes, such as TTN, MUC16, and RYR2, all previously reported to regulate tumorigenesis and chemoresistance in lung cancer, were the most common mutations in both cohorts [24, 25], indicating a lack of significant immune infiltration. Cohort summary plots displayed the distribution of variants according to variant classification type, variant type, and SNV class, identifying missense, SNP, and C>A mutations as the most common (Figure 7(c)). Moreover, top 10 mutated genes were revealed to be *KRAS*, *XIRP2*, *ZFHX4*, *USH2A*, *TP53*, *LRP1B*, *CSMD3*, *RYR2*, *MUC16*, and *TNT* (Figure 7(c)).

### 3.4. Identification of DEGs with LinkedOmics

**Figure 8:**
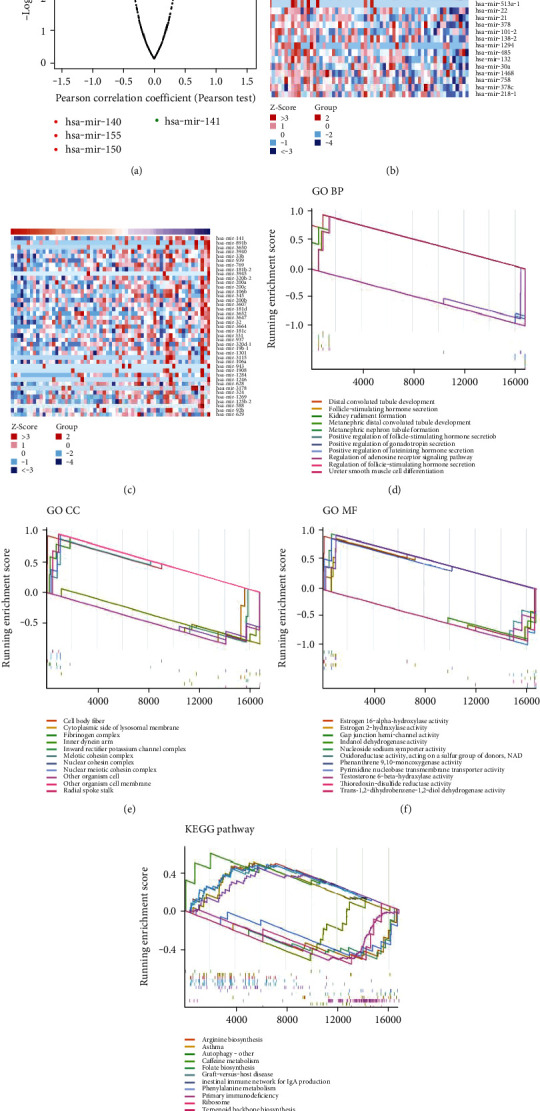
Volcano plot, heatmap, and enrichment analysis of GO and KEGG. (a) Volcano plot. (b, c) Heatmap of upregulated and downregulated genes. (d–g) GO and KEGG enrichment analysis for DEGs, terms with *p* and *q* < 0.05 were believed to be enriched significantly. The green and red dots represent the significantly downregulated and upregulated genes, respectively, and the gray dots represent the genes without differential expression. FDR < 0.05, |log2 FC| > 1, and *p* < 0.05.

A total of 586 DEGs were identified using LinkedOmics, with 141 downregulated and 445 upregulated genes (Figure 8(a)). Heatmaps were constructed to visualize negatively and positively related genes (Figures 8(b) and 8(c)). Subsequent enrichment analysis exhibited a strong correlation between DEGs with immune-related processes, such as graft versus host disease, IgA production, which were further confirmed to be dominant in the TME (Figures 8(d)–8(g)). In addition, pathways related with hormone function and development of kidney and reproduction system were enriched. Notably, the main functions of these DEGs were predominant development and endocrine and immunomodulatory processes, suggesting that the immune component may interact with several pathophysiological factors and play an important role in LUAD development.

### 3.5. Relationship between GIMAP4 Expression and Immune Infiltration

**Figure 9:**
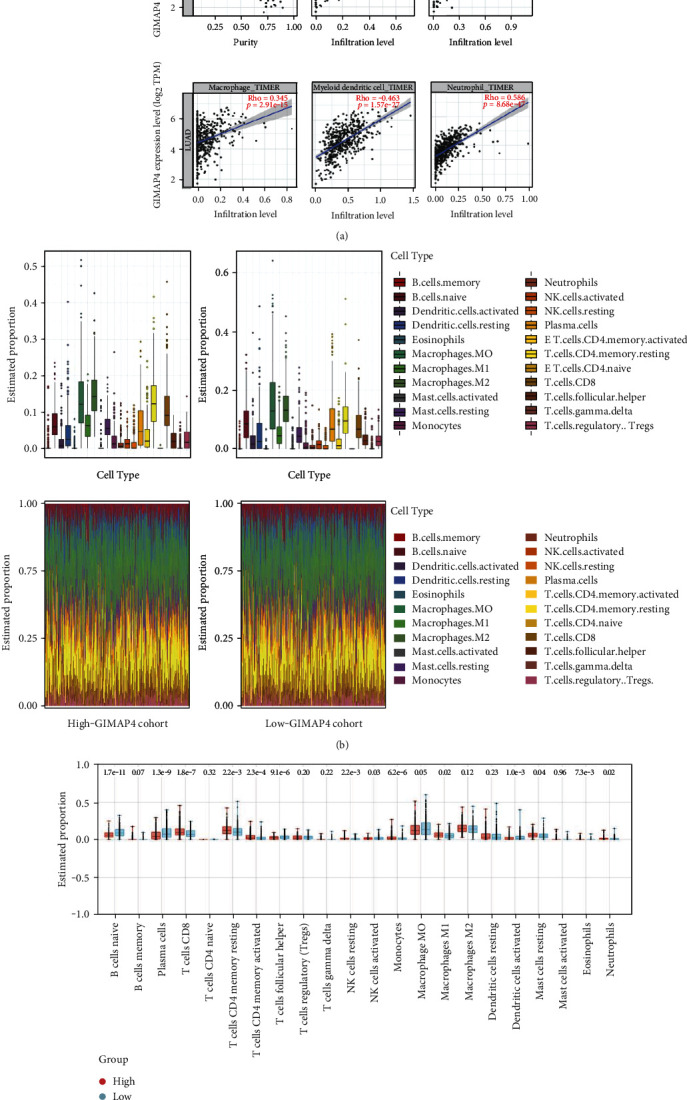
TICs profile in LUAD patients. (a) Relationship between GIMAP4 expression and the different subsets of immune cell infiltrates in lung adenocarcinoma (LUAD) patients using the TIMER database. (b) Bar plot and box plot show the proportion of 22 types of TICs in LUAD samples. (c) Boxplot of immune cell proportion, respectively, in high-GIMAP4 cohort and low-GIMAP4 cohort.

In order to confirm the direct relationship between GIMAP4 expression and immune infiltration, with the exception of B cells and dendritic cells in LUAD patients, a strong positive correlation was observed between GIMAP4 expression and immune cell upregulation (CD4+T cells, CD8+T cells, macrophages, myeloid dendritic cells, and neutrophils) (Figure 9(a)). These findings implied that GIMAP4 was closely intertwined with immune cells in lung cancer. CIBERSORT was used to further confirm the relationship between GIMAP4 expression and the immune components (Figure 9(b)). In both high- and low-GIMAP4 groups of LUAD patients, macrophages, plasma cells, CD4+T cells, and CD8+T cells were the major infiltrating immune cells. Differences in immune cell composition between high-GIMAP4 cohort and low-GIMAP4 cohort were visualized (Figure 9(c)), similar results among CD4+T memory resting cells, CD8+T cells, macrophages, and neutrophils between high- and low-GIMAP4 groups, indicating that high GIMAP4 expression was positively correlated with immune cell infiltration.

**Figure 10:**
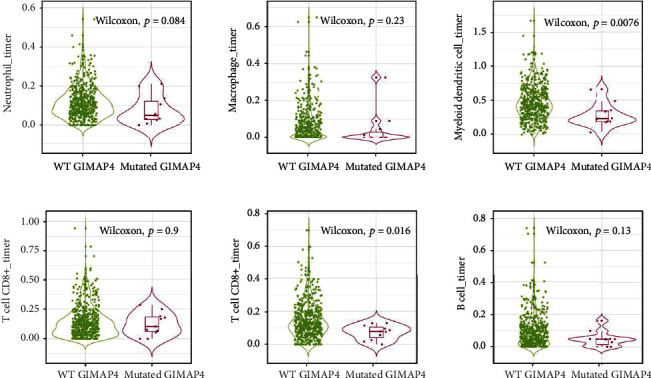
Changes of immune infiltration between tumors with GIMAP4 mutated and WT tumors without mutation.

Likewise, ESTIMATE immune score were also employed to sort samples, revealing significant differences in immune cell composition according to the tumor immune microenvironment (Figure S1). LUAD patients with mutated GIMAP4 had significantly lower infiltrations of neutrophils, myeloid dendritic cells, CD4+ T cells, and B cells, suggesting some disparity in immune response between mutated and wild-type patients (Figure 10).

**Figure 11:**
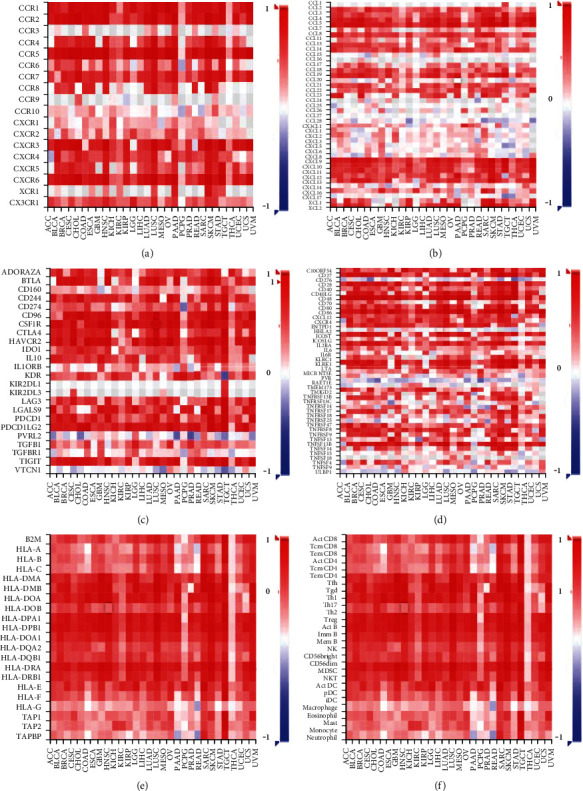
Heatmaps. Spearman correlation between GIMAP4 expression and (a) receptors, (b) chemokine, (c) immunoinhibitor, (d) immunostimulator, (e) MHC molecule, and (f) lymphocyte.

Additionally, heatmaps of spearman correlations between GIMAP4 expression and receptors, chemokines, immunoinhibitors, immunostimulators, MHC molecules, and lymphocytes showed that GIMAP4 plays a vital role in cancer immunomodulation (Figures 11(a)–11(f)).

By contrast, there was no close relationship between immune infiltration level and different sCNA status of GIMAP4 (Figure S2).

**Figure 12:**
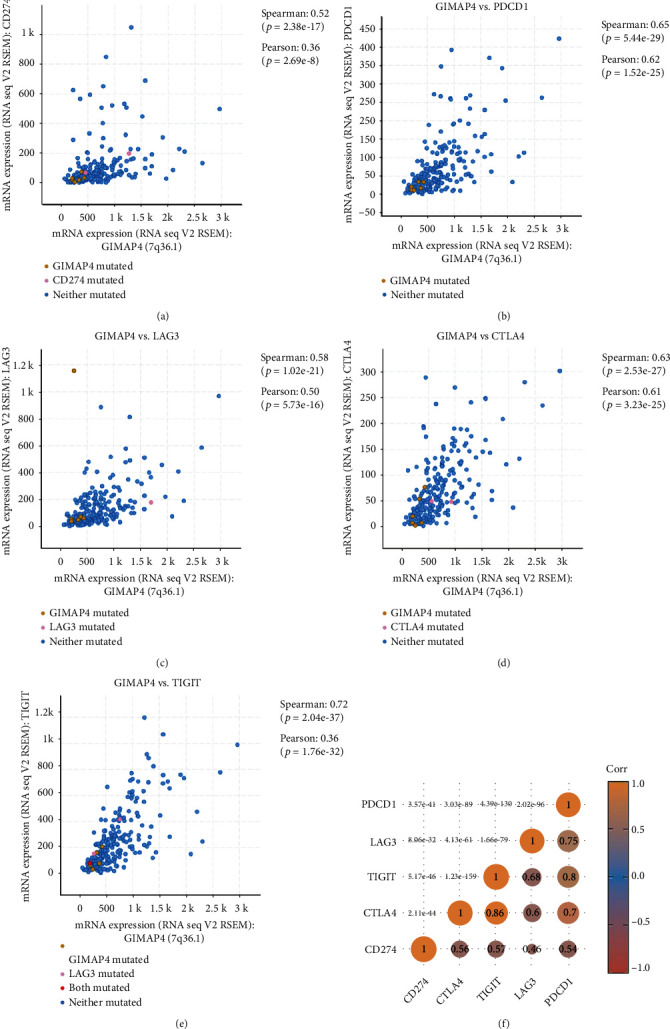
Correlation between GIMAP4 and immune checkpoints (ICPs). (a) GIMAP4 with CD274 (PDL1) in lung adenocarcinoma (LUAD) patients. (b) EGFR with PDCD1 (PD1) in LUAD patients. (c) GIMAP4 with LAG3 in lung adenocarcinoma (LUAD) patients. (d) GIMAP4 with CTLA4 in lung adenocarcinoma (LUAD) patients. (e) GIMAP4 with TIGIT in lung adenocarcinoma (LUAD) patients. (f) A heatmap of the correlation between GIMAP4 and ICPs. The horizontal and vertical coordinates represent genes. The different colors represent correlation coefficients (in the diagram, blue represents positive correlation; red represents negative correlation), and the darker the color represents the two stronger correlations. Asterisks represent levels of significance (^∗^*p* < 0.05, ^∗∗^*p* < 0.01).

Associations between GIMAP4 and ICPs was further probed with pheatmap. Resulting correlation matrices demonstrate that GIMAP4 was positively correlated with CD274 (PDL1), PDCD1 (PD1), CTLA4, LAG3, and TIGIT expression in LUAD, illustrating the potential importance of GIMAP4 in immunotherapies targeting common ICPs (Figure 12).

## 4. Discussion

Among estimated number of incident cases and deaths worldwide, lung cancer remained second according to GLOBOCAN epidemiological statistics in 2020. Lung adenocarcinoma is the most prevalent lung cancer subtype, and previous therapies have not slowed down the continuous increase for incidence and deaths combined. Thus, early lung cancer screening will promote to the direction of a more comprehensive continuous advance of the cancer cure. Our study first linked expression of GIMAP4 in lung cancer tissues with immune components of TME and provide insight into potential GIMAP4-based prognosis prediction. In this paper, we checked differential expression state of GIMAP4 among different tumor types and further found that GIMAP4 matters in the pathogenesis, development, and prognosis of LUAD. Then, we separated LUAD samples from TCGA database into GIMAP4 high-expression and low-expression cohorts in the following procedures. Accordingly, 586 DEGs were subjected to GO and KEGG analysis, which revealed these genes were interacted with TME remodeling: intestinal immune network for IgA production, primary immunodeficiency. From ROC curve, we found that GIMAP4 alone can predict the short-term survival of patients to a certain extent. However, there is a lack of subgroup analysis, and a multicenter cohort study on the prediction effect of GIMAP4 is suggested. According to immunocyte infiltration analysis, GIMAP4 differentiates immunocyte subtypes in TME among samples, such as B lymphocytes, T lymphocytes, monocytes/macrophages, and eosinophils. Constant work should be devoted to give insight into interaction between TME and other physiological processes in LUAD.

Increasing studies have indicated that the TME plays a critical role in the carcinogenesis and development of cancer through immunocyte subtype composition remodeling. In current immunotherapeutic area, identification of a mediator capable of transforming the TME from a tumor-friendly to a suppressive environment is urgently needed. Notably, studies revealed that GIMAPs are preferentially expressed in immune cells with several GIMAP family members being involved in the development of lymphocytes [26]. Consequently, GIMAPs are implicated in the development of T-lymphopenia, leukemia, and autoimmunity by further interaction with Bcl-2 family proteins [27]. So far, GIMAPs have been recognized performing a wide range of functions such as thymocyte development, apoptosis of peripheral lymphocytes, and T helper cell activation. Deficiency or mutation of these genes would be a strong risk factor regarding diverse immunological diseases. For instance, *GIMAP3* and *GIMAP5* gene knockout mice presented development and maturation defection of the thymus [28–30]. Considering its immune system regulating function, GIMAP4 might be a potential cancer suppressor target. Indeed, Mégarbané et al. once raised the hypothesis [13], now confirmed both clinically and *in silico*, suggesting GIMAP4 as an accelerator of programmed cell death [31].

A common feature of various tumors is their ability to escape the host immune response by secreting Th2 cytokines which favor an immune-suppressed TME [32]. Inversely, the dominant state of Th1 cytokines suppresses tumor growth, metastasis, and drug-resistance, in some cases inducing tumor regression [33]. As a result, Th1/Th2 ratio determines immunotherapeutic effect and regulatory factors of Th1/Th2 shift are potential pharmaceutical target. Recent research found that cancer immunotherapies employing patients' individualized TICs effectively treated NSCLC, albeit without differential effects [34]. Additionally, previous studies have proven that GIMAP4 is capable of generating tumor-specific neoantigens and activating the immune system [35, 36]. Therefore, Xu et al. [12] came to a conclusion that GIMAP4 reversed the Th1/Th2 drifting effect and enhanced the immunity of Th1. Recently, evidence indicated *IL-12* as a promising target for antitumor immunotherapy. IL-12 is a proinflammatory cytokine composed of p40 and p35 subunits [37]. IL-12 derives from antigen-presenting cells, such as dendritic cells and macrophages, and is crucial for the recruitment of immune killer cells [38]. Moreover, it was verified essential in the differentiation of the Th1 lineage and was found to upregulate GIMAP4 and Th1 cytokines as well. This is corroborated by our immune cell components analysis; parallel alteration existed in T cell subgroup proportion and GIMAP4 expression. Then, enrichment analysis consolidated this hypothesis, wherein we demonstrated an involvement of cytoplasmic component and cytoskeleton function alteration in LUAD. A positive feedback loop may exist between IL-12 and GIMAP4, providing a potential target of novel therapies. Taken together, a primitive therapeutic strategy could be: (a) directly inject Th1 cytokines into the body; (b) gene therapy with the above cytokines; (c) inject anti-Th2 cytokine antibody to enhance Th1/Th2 shift; (d) active immunotherapy to bolster classical immune response pathways.

Cancer cells have been confirmed to disguise tumor-specific antigens and escape immunological surveillance via ICP pathways. A myriad of research has focused on immune checkpoint inhibitors, mostly displaying a solid antitumor effect against a broad spectrum of cancer types [39, 40]. Case in point, Zhang et al. [41] carried out treatment directly targeting ICPs, achieving tremendous success in the history of anticancer worldwide. In this situation, we hypothesized that GIMAP4 may trigger ICP-related response. To explore mechanism of GIMAP4 underlying tumor-immune regulation, we calculated the correlations between GIMAP4 and 5 ICPs (CD274, CTLA4, TIGIT, LAG3, and PDCD1) and identified similar trends in their expression. This result provides theoretical support for ICP treatment based on GIMAP4 expression-grouped LUAD patients. Clinically, treatment targeting aforementioned ICPs in lung cancer exhibited significant efficacy in certain studies [42–44]. LAG3 and TIGIT, two classical ICPs, were detected in tumor-infiltrating lymphocytes and showed a prominent association with other ICPs. In 90 samples treated with PD-1 axis blockers, high LAG3 was associated with worse prognosis [45, 46]. Similar finding was reported on TIGIT in another study [47]. We expect to see further immunotherapies targeting anti-LAG3 and anti-TIGIT pathways in the future. Simultaneously, we hope that the specific mechanism between GIMAP4 and ICI therapeutic strategies will be further investigated and elucidated.

Since elevated ICP expression was observed in high-GIMAP4 expressing group, GIMAP4 was probed as an intrinsic resistance factor to immunotherapy. Nevertheless, therapeutic strategies based on immune checkpoints inhibitors, alone or in combinations, at present seemed to be insufficient in prompting tumor regression in a large number of patients across a broad spectrum of advanced solid cancers, due to the presence of intrinsic and acquired resistance. Additionally, current studies prevailingly concentrate on gene expression profile or somatic mutation data, which placed limitations on our exploration into deeper mechanisms. Furthermore, our research lacks clinical or wet-lab components and mainly focuses on public databases. As mentioned above, our research only focused on GIMAP4, while GIMAP4 was reported to participate in a wide range of biological function and interact with other factors. Integration of more related genes may strengthen its prediction potential. In addition, GIMAP4 expression varies among patients. There is a lack of subgroup analysis on the effect of GIMAP4-based ROC curve. We will further conduct a study on GIMAP4 to clarify its predictive value with multiple clinical cohorts, based on multiple cox regression analysis and multifactor ROC curves.

## 5. Conclusions

In this study, GIMAP4 was identified as a promising index for predicting immune responses, which was also observed as a prognostic biomarker in clinical outcomes, including overall survival. Further studies should be conducted to clarify the relationship between GIMAP4 and ICPs with Th1/Th2, which may benefit LUAD patients.

## Data Availability

The data generated in this work can be accessed from the public database.

## Supplementary Materials

Supplementary Materials Supplementary Figure 1: the differential proportion of 22 types of TICs between high and low immune score groups of LUAD samples. Supplementary Figure 2: correlation between immune infiltration level and different sCNA status of GIMAP4 on violin plots.

## References

[1] Duma N., Santana-Davila R., Molina J. R. (2019). Non-small cell lung cancer: epidemiology, screening, diagnosis, and treatment. *Mayo Clinic Proceedings*.

[2] Bade B. C., Dela Cruz C. S. (2020). Lung cancer 2020: epidemiology, etiology, and prevention. *Clinics in Chest Medicine*.

[3] Barta J. A., Powell C. A., Wisnivesky J. P. (2019). Global epidemiology of lung cancer. *Annals of Global Health*.

[4] Bremnes R. M., Busund L. T., Kilvaer T. L. (2016). The role of tumor-infiltrating lymphocytes in development, progression, and prognosis of non-small cell lung cancer. *Journal of Thoracic Oncology*.

[5] Lee S. S., Cheah Y. K. (2019). The interplay between microRNAs and cellular components of tumour microenvironment (TME) on non-small-cell lung cancer (NSCLC) progression. *Journal of Immunology Research*.

[6] Wang S. S., Liu W., Ly D., Xu H., Qu L., Zhang L. (2019). Tumor-infiltrating B cells: their role and application in anti-tumor immunity in lung cancer. *Cellular & Molecular Immunology*.

[7] Gardner A., Ruffell B. (2016). Dendritic cells and cancer immunity. *Trends in Immunology*.

[8] Guo X., Zhang Y., Zheng L. (2018). Global characterization of T cells in non-small-cell lung cancer by single- cell sequencing. *Nature Medicine*.

[9] Kagabu M., Nagasawa T., Sato C. (2020). Immunotherapy for uterine cervical cancer using checkpoint Inhibitors: Future Directions. *International Journal of Molecular Sciences*.

[10] Lin A., Wei T., Meng H., Luo P., Zhang J. (2019). Role of the dynamic tumor microenvironment in controversies regarding immune checkpoint inhibitors for the treatment of non-small cell lung cancer (NSCLC) with EGFR mutations. *Molecular Cancer*.

[11] Heinonen M. T., Laine A.-P., Söderhäll C. (2015). GIMAP GTPase family genes: potential modifiers in autoimmune diabetes, asthma, and allergy. *Journal of Immunology*.

[12] Xu F., Shen J., Xu S. (2021). Integrated bioinformatical analysis identifies GIMAP4 as an immune-related prognostic biomarker associated with remodeling in cervical cancer tumor microenvironment. *Frontiers in Cell and Developmental Biology*.

[13] Megarbane A., Piquemal D., Rebillat A. S. (2020). Transcriptomic study in women with trisomy 21 identifies a possible role of the GTPases of the immunity-associated proteins (GIMAP) in the protection of breast cancer. *Scientific Reports*.

[14] Shiao Y. M., Chang Y. H., Liu Y. M. (2008). Dysregulation of GIMAP genes in non-small cell lung cancer. *Lung Cancer*.

[15] Buckley A. R., Ideker T., Carter H., Harismendy O., Schork N. J. (2018). Exome-wide analysis of bi-allelic alterations identifies a Lynch phenotype in The Cancer Genome Atlas. *Genome Medicine*.

[16] Selamat S. A., Chung B. S., Girard L. (2012). Genome-scale analysis of DNA methylation in lung adenocarcinoma and integration with mRNA expression. *Genome Research*.

[17] Boelens M. C., Gustafson A. M., Postma D. S. (2011). A chronic obstructive pulmonary disease related signature in squamous cell lung cancer. *Lung Cancer*.

[18] Mayakonda A., Lin D. C., Assenov Y., Plass C., Koeffler H. P. (2018). Maftools: efficient and comprehensive analysis of somatic variants in cancer. *Genome Research*.

[19] Vasaikar S. V., Straub P., Wang J., Zhang B. (2018). LinkedOmics: analyzing multi-omics data within and across 32 cancer types. *Nucleic Acids Research*.

[20] Li T., Fu J., Zeng Z. (2020). TIMER2.0 for analysis of tumor-infiltrating immune cells. *Nucleic Acids Research*.

[21] Ritchie M. E., Phipson B., Wu D. (2015). Limma powers differential expression analyses for RNA-sequencing and microarray studies. *Nucleic Acids Research*.

[22] Newman A. M., Liu C. L., Green M. R. (2015). Robust enumeration of cell subsets from tissue expression profiles. *Nature Methods*.

[23] Ru B., Wong C. N., Tong Y. (2019). TISIDB: an integrated repository portal for tumor-immune system interactions. *Bioinformatics*.

[24] Luo J., Liu Z. (2019). Long non-coding RNA TTN-AS1 promotes the progression of lung adenocarcinoma by regulating PTEN/PI3K/AKT signaling pathway. *Biochemical and Biophysical Research Communications*.

[25] Kanwal M., Ding X. J., Song X., Zhou G. B., Cao Y. (2018). MUC16 overexpression induced by gene mutations promotes lung cancer cell growth and invasion. *Oncotarget*.

[26] Ciucci T., Bosselut R. (2014). Gimap and T cells: a matter of life or death. *European Journal of Immunology*.

[27] Nitta T., Takahama Y. (2007). The lymphocyte guard-IANs: regulation of lymphocyte survival by IAN/GIMAP family proteins. *Trends in Immunology*.

[28] Yano K., Carter C., Yoshida N. (2014). Gimap3 and Gimap5 cooperate to maintain T-cell numbers in the mouse. *European Journal of Immunology*.

[29] Schulteis R. D., Chu H., Dai X. (2008). Impaired survival of peripheral T cells, disrupted NK/NKT cell development, and liver failure in mice lacking Gimap5. *Blood*.

[30] Barnes M. J., Aksoylar H., Krebs P. (2010). Loss of T cell and B cell quiescence precedes the onset of microbial flora-dependent wasting disease and intestinal inflammation in Gimap5-deficient mice. *Journal of Immunology*.

[31] Anders S., Huber W. (2010). Differential expression analysis for sequence count data. *Genome Biology*.

[32] Kidd P. (2003). Th1/Th2 balance: the hypothesis, its limitations, and implications for health and disease. *Alternative Medicine Review: A Journal of Clinical Therapeutic*.

[33] Dai M., Hellstrom I., Yip Y. Y., Sjogren H. O., Hellstrom K. E. (2018). Tumor regression and cure depends on sustained Th1 responses. *Journal of Immunotherapy*.

[34] Guo X., Zhang Y., Zheng L. (2018). Publisher correction: global characterization of T cells in non-small-cell lung cancer by single-cell sequencing. *Nature Medicine*.

[35] Heinonen M. T., Kanduri K., Lahdesmaki H. J., Lahesmaa R., Henttinen T. A. (2015). Tubulin- and actin-associating GIMAP4 is required for IFN-*γ* secretion during Th cell differentiation. *Immunology and Cell Biology*.

[36] Filen J. J., Filen S., Moulder R. (2009). Quantitative proteomics reveals GIMAP family proteins 1 and 4 to be differentially regulated during human T helper cell differentiation. *Molecular & Cellular Proteomics*.

[37] Mirlekar B., Pylayeva-Gupta Y. (2021). IL-12 family cytokines in cancer and immunotherapy. *Cancers*.

[38] Zundler S., Neurath M. F. (2015). Interleukin-12: functional activities and implications for disease. *Cytokine & Growth Factor Reviews*.

[39] Bagchi S., Yuan R., Engleman E. G. (2021). Immune checkpoint inhibitors for the treatment of cancer: clinical impact and mechanisms of response and resistance. *Annual Review of Pathology*.

[40] Singh S., Hassan D., Aldawsari H. M., Molugulu N., Shukla R., Kesharwani P. (2020). Immune checkpoint inhibitors: a promising anticancer therapy. *Drug Discovery Today*.

[41] Zhang Z., Bao S., Yan C., Hou P., Zhou M., Sun J. (2021). Computational principles and practice for decoding immune contexture in the tumor microenvironment. *Briefings in Bioinformatics*.

[42] Chae Y. K., Arya A., Iams W. (2018). Current landscape and future of dual anti-CTLA4 and PD-1/PD-L1 blockade immunotherapy in cancer; lessons learned from clinical trials with melanoma and non-small cell lung cancer (NSCLC). *Journal for Immunotherapy of Cancer*.

[43] Forde P. M., Chaft J. E., Smith K. N. (2018). Neoadjuvant PD-1 blockade in resectable lung cancer. *The New England Journal of Medicine*.

[44] Constantinidou A., Alifieris C., Trafalis D. T. (2019). Targeting programmed cell death -1 (PD-1) and ligand (PD-L1): a new era in cancer active immunotherapy. *Pharmacology & Therapeutics*.

[45] Datar I., Sanmamed M. F., Wang J. (2019). Expression analysis and significance of PD-1, LAG-3, and TIM-3 in human non-small cell lung cancer using spatially resolved and multiparametric single-cell analysis. *Clinical Cancer Research*.

[46] He Y., Yu H., Rozeboom L. (2017). LAG-3 protein expression in non-small cell lung cancer and its relationship with PD-1/PD-L1 and tumor-infiltrating lymphocytes. *Journal of Thoracic Oncology*.

[47] Hu F., Wang W., Fang C., Bai C. (2020). TIGIT presents earlier expression dynamic than PD-1 in activated CD8^+^ T cells and is upregulated in non-small cell lung cancer patients. *Experimental Cell Research*.

