# Supplementary figures and images for: Prognostic Value of GIMAP4 and Its Role in Promoting Immune Cell Infiltration into Tumor Microenvironment of Lung Adenocarcinoma
Source: Biomed Res Int. 2022 Oct 6;2022:7440189. doi: 10.1155/2022/7440189 (PMC9560834; doi:10.1155/2022/7440189)

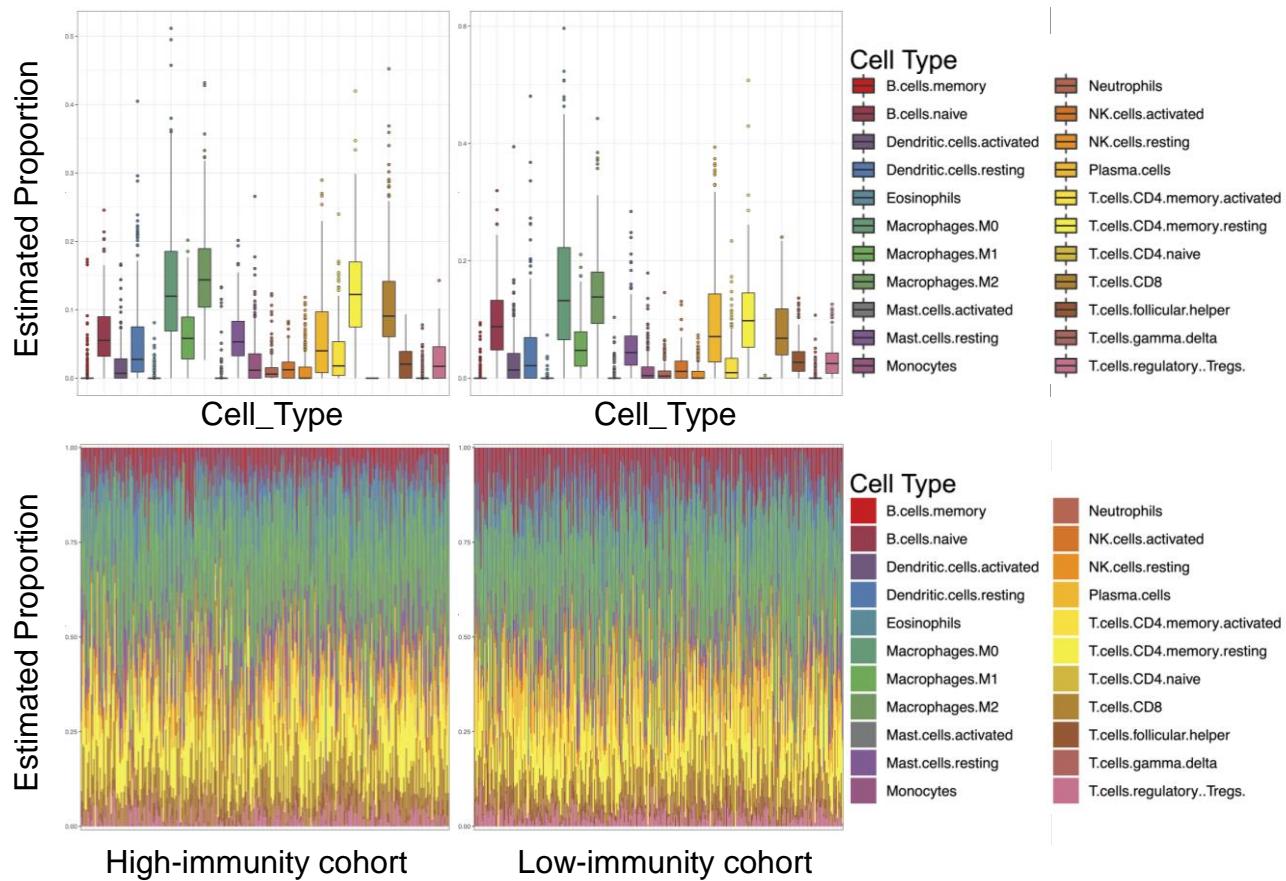

Supplement: Supplementary Materials — Supplementary Figure 1: the differential proportion of 22 types of TICs between high and low immune score groups of LUAD samples. Supplementary Figure 2: correlation between immune infiltration level and different sCNA status of GIMAP4 on violin plots. [file 7440189.f1.zip › figure s1 (1).pdf]

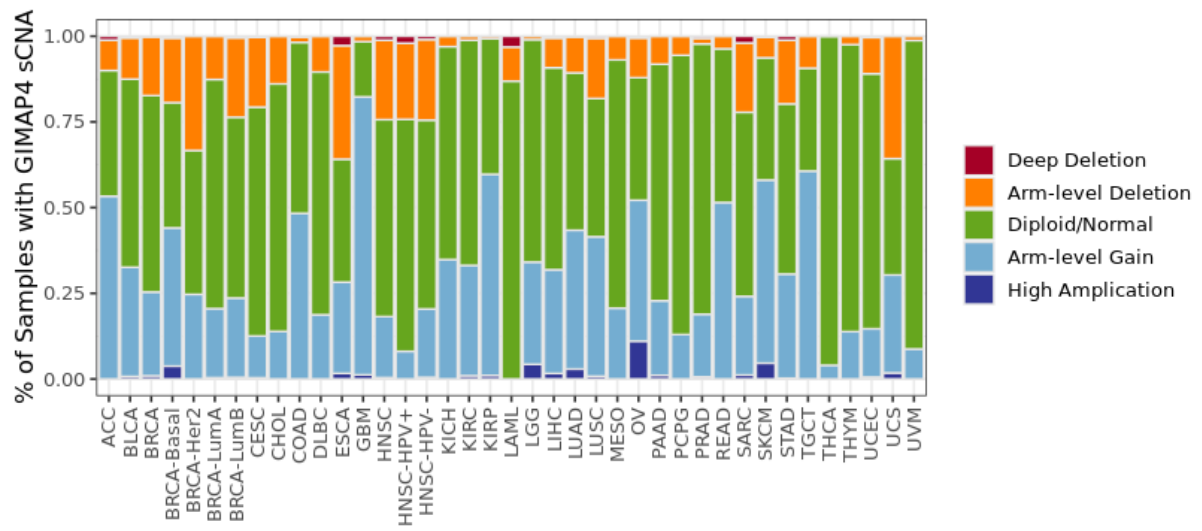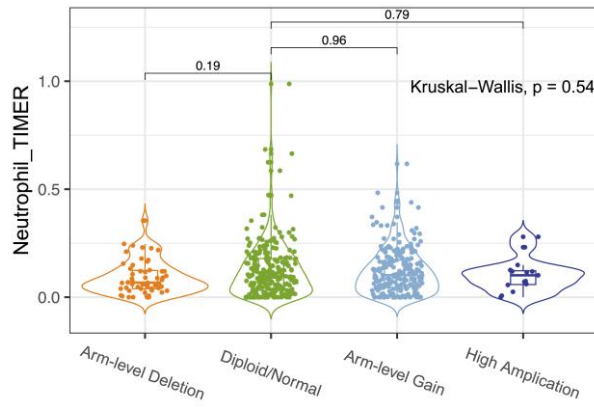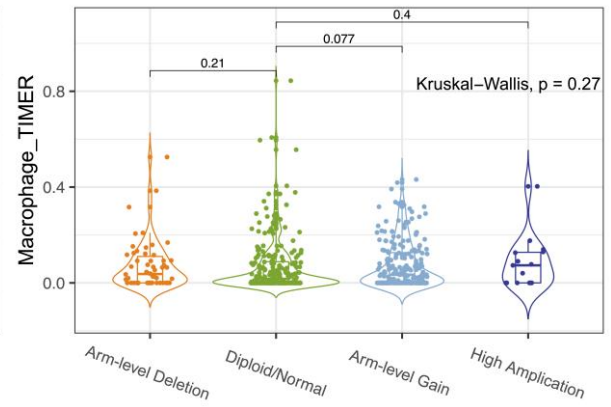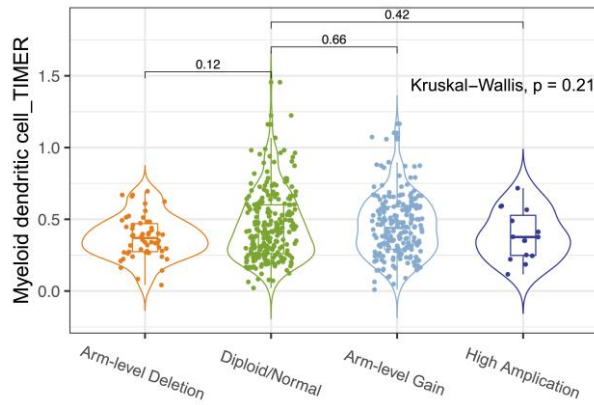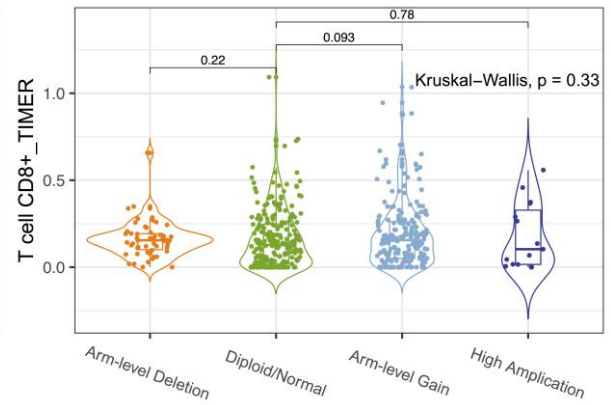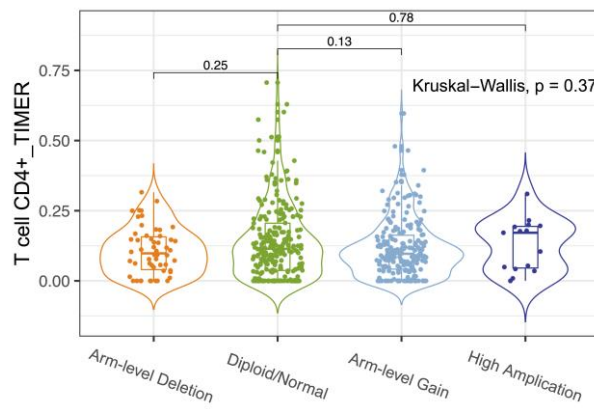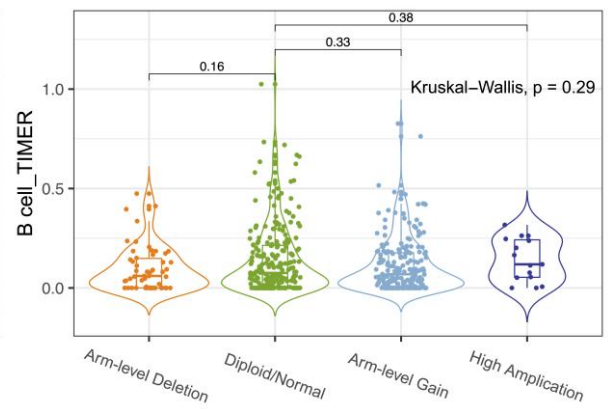

Supplement: Supplementary Materials — Supplementary Figure 1: the differential proportion of 22 types of TICs between high and low immune score groups of LUAD samples. Supplementary Figure 2: correlation between immune infiltration level and different sCNA status of GIMAP4 on violin plots. [file 7440189.f1.zip › figure s2 (1).pdf]
